# CRISPR-Based COVID-19 Testing: Toward Next-Generation Point-of-Care Diagnostics

**DOI:** 10.3389/fcimb.2021.663949

**Published:** 2021-04-30

**Authors:** Uyanga Ganbaatar, Changchun Liu

**Affiliations:** ^1^ Department of Biomedical Engineering, University of Connecticut Health Center, Farmington, CT, United States; ^2^ Department of Biomedical Engineering, University of Connecticut, Storrs, CT, United States

**Keywords:** COVID-19, coronavirus, SARS-CoV-2, CRISPR-based nucleic acid detection, point-of-care testing

## Abstract

As the COVID-19 pandemic continues, people are becoming infected at an alarming rate, individuals are unknowingly spreading disease, and more lives are lost every day. There is an immediate need for a simple, rapid, early and sensitive point-of-care testing for COVID-19 disease. However, current testing approaches do not meet such need. Recently, clustered regularly interspaced short palindromic repeats (CRISPR)-based detection methods have received substantial attention for nucleic acid-based molecular testing due to their simplicity, high sensitivity and specificity. This review explores the various CRISPR-based COVID-19 detection methods and related diagnostic devices. As with any emerging technology, CRISPR/Cas-based nucleic acid testing methods have several challenges that must be overcome for practical applications in clinics and hospitals. More importantly, these detection methods are not limited to COVID-19 but can be applied to detect any type of pathogen, virus, and fungi that may threaten humans, agriculture, and food industries in resource-limited settings. CRISPR/Cas-based detection methods have the potential to become simpler, more reliable, more affordable, and faster in the near future, which is highly important for achieving point-of-care diagnostics.

## Introduction

The relatively recent outbreak of coronavirus disease 2019 (COVID-19) in Wuhan, China has now become a global pandemic. COVID-19 is caused by severe acute respiratory syndrome coronavirus-2 (SARS-CoV-2), better known as coronavirus. Yet, SARS-CoV-2 is not the only virus that has recently emerged. Although coronavirus is the most recent case, one virus has emerged each year (Howard and Fletcher, 2012), threatening public health, the global economy, and even personal freedom. The spread of coronavirus primarily occurs *via* local community transmission (Liu et al., 2020), and this global pandemic has shown that most countries were not prepared for an outbreak due to a lack of rapid, reliable, point-of-care detection and treatment, which has enabled further spreading of the virus. As of February 2021, more than 102 million people around the globe have been infected, with over two million deaths due to this virus (“WHO Coronavirus Disease (COVID-19) Dashboard | WHO Coronavirus Disease (COVID-19) Dashboard” n.d.). Thus, there is an immediate need for simple, rapid, and affordable testing for every facility around the world, from developing to first-world nations.

Coronaviruses are single-stranded RNA (ssRNA) viruses that belong to the family of *Coronaviridae.* These viruses have eight accessory proteins and four major structural proteins. Different laboratories around the world have targeted different components of SARS-CoV-2 to enable its detection, including the spike protein (S), small envelope protein (E), nucleocapsid protein (N), and RNA-dependent RNA polymerase (RdRp) gene of the ORF1ab sequence (Wang H. et al., 2020).

Numerous detection methods exist for COVID-19, including molecular, antigen, and antibody tests, each with their own benefits and drawbacks (“Coronavirus Testing Basics | FDA” n.d.). Currently, the most common detection method is the molecular test, also known as the nucleic acid detection, which determines whether patient sample (e.g., nasopharyngeal swab, saliva) has SARS-CoV-2 genetic material (Lisboa Bastos et al., 2020). To perform this test, viral RNA is first isolated and extracted from patient samples. Then, the purified RNA is subjected to reverse transcription quantitative polymerase chain reaction (RT-qPCR), which converts the virus RNA to DNA and amplifies the DNA to produce millions of copies. Finally, a detection probe can determine whether the patient has viral genetic material (“Coronavirus Testing Basics | FDA” n.d.). While the RT-PCR method is considered as the gold standard method, it does have some drawbacks. For instance, this test can produce false negative results (Chen Z. et al., 2020), there are shortages of test kits (American Society of Microbiology, 2020), and a period of several hours to few days is needed to obtain results. Moreover, this method requires expensive equipment, a molecular laboratory, and a trained scientist (Rao et al., 2020). Having a simple, reliable point-of-care testing (POCT) method will help physicians and patients fill in the gaps of our current testing for SARS-CoV-2.

**Figure 1:**
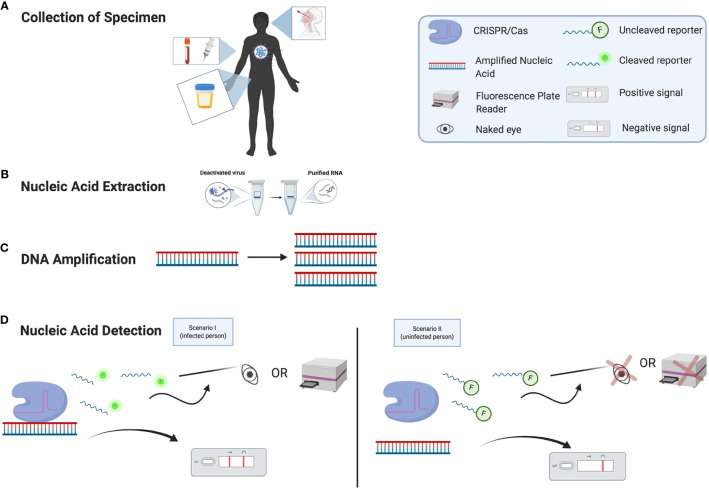
Nucleic Acid Detection of SARS-CoV-2 Using CRISPR/Cas Assays. **(A)** Patient specimens can be collected from different types of clinical samples. **(B)** RNA is extracted from the specimen. **(C)** From the nucleic acid extraction, the DNA must be amplified. **(D)** The nucleic acid of SARS-CoV-2 can now be detected. If a person has COVID-19 (Scenario I), then the CRISPR/Cas complex will bind to the target region of the amplified nucleic acid and collateral cleavage activity can occur by cleaving the nearby fluorescence reporter nucleic acids. This can be detected by either by using the naked eye under specific light, a fluorescence plate reader, or a lateral flow assay that can indicate the presence of the virus’s nucleic acid. If a person does not have COVID-19 (Scenario II), then the CRISPR/Cas complex will not bind to the target region of the amplified nucleic acid and collateral cleavage activity will not be initiated; this means that there will not be any viral signal (glow) from the sample observed by the naked eye, a plate reader, or a lateral flow assay.

Recently, clustered regularly interspaced short palindromic repeats (CRISPR), has received substantial attention for nucleic acid detection due to its simplicity, speed, high sensitivity and specificity. With the help of CRISPR RNA (crRNA), CRISPR proteins can specifically cut the target region that is complemented with the crRNA sequence. There are two components in the CRISPR detection: first, the CRISPR-RNA complex will cut the target region, this activates the next step, collateral cleavage of the surrounding nucleic acids. CRISPR Cas12, Cas13, and Cas14 effector proteins have a unique collateral cleavage ability, which enables these tools to indiscriminately cleave surrounding nucleic acid once they bind to the target site (**Figure 1**). With regard to cleavage activity, Cas12 recognizes double-stranded DNA (dsDNA) more efficiently than single-stranded DNA (ssDNA), but it still exhibits collateral activity for ssDNA. Cas14 recognizes ssDNA more effectively than dsDNA with respect to cleavage activity and also exhibits collateral activity for ssDNA. Cas13 is unique because it recognizes and exhibits collateral activity for ssRNA (van Dongen et al., 2020). By introducing appropriate nucleic acid reporters (ssDNA, ssRNA, dsDNA), different detection signals (e.g., fluorescent, colorimetric, electrochemical) can be specifically detected. Because of this collateral cleavage activity, CRISPR can be combined with isothermal nucleic acid amplification to simplify the detection method by visualizing the result of positive or negative samples with the naked eye, LED or UV lamps, or by observing the lateral flow strips. Traditional methods like PCR or RT-qPCR need expensive, bulky equipment, especially PCR needs to either run on an agarose gel for visualization of the target DNA. This takes longer, requires labor-intensive work and opening the amplified tube increases the contamination rate. Moreover, isothermal amplification like recombinase polymerase amplification (RPA) and loop-mediated isothermal amplification (LAMP) alone is not specific enough and single-nucleotide polymorphisms (SNPs) cannot be discriminated (van Dongen et al., 2020). However, combination of isothermal amplification and CRISPR improves the sensitivity and specificity because the crRNA only binds to the target region and can even detect SNPs for human genotyping (Gootenberg et al., 2017).

POCT methods can rapidly generate results at a location close to the patient (Vashist, 2017), which reduces the duration for which a patient must stay at a clinic. Demand for POCT has increased over the last 40 years (St John and Price, 2014) and is especially high today because of the current global pandemic. Popular POCT technologies, such as glucose biosensor strips and lateral flow assays, have been widely used for home healthcare and disease detection. These devices eliminate the need to return to the doctor’s office multiple times, thus saving the patient money (St John and Price, 2014), which is especially important in countries without affordable healthcare. These devices are also extremely beneficial for remote regions and cities that do not possess extensive, costly diagnostic tools (Tideman et al., 2014). In particular, there is substantial interest in integrating CRISPR-based molecular testing into lateral flow detection and microfluidic technology, enabling simple, rapid and reliable point-of-care diagnostics.

In this review, we focus on CRISPR-based diagnostic tools as well as their benefits and drawbacks. We review CRISPR as a nucleic acid detection tool for SARS-CoV-2 and report on research efforts occurring worldwide to improve the specificity, sensitivity, and speed of methods for detecting this virus. For those tools that are not ready for POCT, we discuss current challenges and future directions.

## CAS12-Effector-Based COVID-19 Diagnostic Tools

The breakthrough of using Cas12a in nucleic acid detection was first reported by Chen et al. who developed DNA endonuclease-targeted CRISPR trans reporter (DETECTR) for human papillomavirus (HPV) in 2018 (Chen J. S. et al., 2018). Due to the discovery of collateral activity in Cas12a, Chen et al. have explored its potential as a diagnostic tool. DETECTR was developed using crRNA and ssDNA fluorophore quencher (ssDNA-FQ) reporter by combining recombinase polymerase amplification RPA with Cas12a-based fluorescence detection. RPA conveniently eliminates the need for a labor-intensive, temperature-sensitive PCR method because RPA operates under isothermal conditions (e.g., ~37°C). With RPA assay, this tool is close to POCT. However, the fluorescence detection by using a fluorescence plate reader is not ideal for POCT applications. Recently, Broughton et al. adapted DETECTR to lateral flow assay for SARS-CoV-2 detection (Broughton et al., 2020). This version utilizes reverse transcription loop-mediated isothermal amplification (RT-LAMP) instead of RPA because the patient samples consist of RNA from nasopharyngeal or oropharyngeal swabs. The RT-LAMP-DETECTR reaction performs well at 62°C, which is less convenient than the RPA-DETECTR for the HPV virus, which worked at 37°C. Broughton et al. designed the crRNA around the N and E gene regions of SARS-CoV-2, because the detection methods utilized by the World Health Organization and US Centers for Disease Control (CDC) and Prevention operate around those regions. The detection results can be obtained more quickly with this method than with previous DETECTR detection methods (30-40 min vs. >1 h). In particular, they employed a lateral flow strip instead of a fluorescence plate reader, bringing this method closer to POCT and eliminating the need for bulky instrumentation. Broughton et al. compared the lateral flow strip method against the currently most commonly used CDC assay to test their limit of detection (LoD). The CDC assay could detect the virus at levels as low as 1 viral copy per *µ*L while DETECTR could detect the virus at levels as low as 10 viral copies per *µ*L using *in vitro* transcribed viral nucleoprotein RNA (Broughton et al., 2020). The authors then tested 11 clinical respiratory samples using this device. One of the samples had a quality control failure, and another gave the wrong result, but the results for the remaining nine samples matched the clinical test results. Thus, the performance is promising, but not perfect. The reason that two of their samples failed is because the samples contained fewer than 10 viral copies per *µ*L. These researchers increased their sample size and compared results for 60 nasopharyngeal swab samples with CDC RT-qPCR assay results and claimed to obtain similar results. Interestingly, their crRNA was designed to target the E gene of SARS-CoV-2 in order to detect other coronavirus strains. But this may influence the detection specificity. The researchers tested the sensitivity of this assay at femtomolar (fM) levels rather than attomolar (aM) levels, as had been previously done. It would be interesting to determine whether aM detection sensitivity is achieved for this specific virus as well.

Several other groups have designed detection methods very similar to DETECTR for SARS-CoV-2. One such method is the CRISPR-based fluorescent detection system (CRISPR-FDS); however, this method requires a micro-plate fluorescent reader, which is not a substantial improvement in terms of POCT (Huang et al., 2020). Another group developed the *in vitro* specific CRISPR-based assay for nucleic acid detection (iSCAN) (Ali et al., 2020). The authors also developed this CRISPR Cas12a-based detection method with RT-LAMP. Their LoD was 10 RNA copies per reaction, which is difficult to compare with that of DETECTR, because these two LoD are in different units. The E gene employed in SARS-CoV-2 detection had a low sensitivity (38% accurate for positive, 100% accurate for negative SARS-CoV-2 samples), while the N gene detection was better (86% accurate for positive, 100% accurate for negative SARS-CoV-2 samples). To bring this method closer to POCT, the authors developed the assay as a one-pot technique. For this purpose, they replaced Cas12a with Cas12b because Cas12b can function at the same temperature as RT-LAMP; however, the efficiency of one-pot assay was low. To this end, they added CRISPR/Cas12b on the tube wall as a droplet, which remained until the RT-LAMP process was completed. They then mixed the sample and finally obtained a similar sensitivity (86% accurate for positive, 100% accurate for negative SARS-CoV-2 samples). Another group developed a similar method by adding mineral oil on top of the RT-LAMP sample to act as a barrier and then added the CRISPR Cas12a reagent to the top of the microcentrifuge tube (Chen Y. et al., 2020). The authors shook the samples by hand after RT-LAMP was performed. Adding CRISPR-Cas12b as a droplet can be risky because if the droplet is not sufficiently large, the CRISPR reagents can evaporate during the 30-min amplification step. In addition, the droplet could slide down and mix with the sample. As an advantage, the likelihood of introducing contamination is lower than cases in which the tube is reopened to add enzymes. However, the efficiency of this assay is not sufficient to replace current RT-qPCR CDC assays.

Two different groups independently developed an improved CRISPR-based detection method to visualize fluorescence signals using the naked eye. This feature is a great advantage because it can eliminate the need for an ultraviolet (UV) light source, which can be bulky and expensive. CRISPR/Cas12a-based detection with the naked eye readout (CRISPR/Cas12a-NER) utilizes reagents similar to those for other methods, with the exception of reagents for reverse-transcription recombinase-aided amplification (RT-RAA) (Wang X. et al., 2020). This method is a two-pot assay because the RT-RAA occurs at 39°C while the CRISPR/Cas12a process operates at 37°C. Conversely, the samples can be visualized under a blue light-emitting diode (LED). Another group developed AIOD-CRISPR (all-in-one dual CRISPR-Cas12a), which can also be visualized under blue LED light or UV light and, in some cases, even under ambient light without excitation (Ding et al., 2020). These researchers used RT-RPA, which occurs at 37°C, along with the CRISPR/Cas12a activity, making this assay a true one-pot approach. More importantly, this method utilizes two crRNAs that are not limited by a protospacer adjacent motif (PAM) site. This is significant because Broughton et al., 2020 stated they could not design crRNAs in the N1 or N3 regions of SARS-CoV-2 due to the lack of suitable PAM site (Broughton et al., 2020). However, Ding et al. demonstrated the feasibility of designing crRNAs without being limited by the PAM site. Additionally, the authors showed that the use of two crRNAs could improve the assay sensitivity (Ding et al., 2020). Ding et al. reported that although the use of PAM-limited crRNA is the fastest method for detecting SARS-CoV-2 plasmid, the combination of two crRNAs that are not limited by the PAM could still be useful for detection. This finding can greatly help scientists in the design of crRNAs for detection methods, indicating that these designs do not need to be restricted by the availability of the PAM site in the targeted region. By eliminating the separate pre-amplification step, the authors created a one-pot assay. Out of 28 clinical samples, this assay detected 8 RNA extracts as positive for SARS-CoV-2. This device needs to be performed on larger, more diverse clinical samples and some improvements on nucleic acid sample preparation are still needed for POCT. Further improvement may be achieved by integrating simple nucleic acid preparation with their AIOD-CRISPR assay, enabling simple, rapid, and “sample to answer” SARS-CoV-2 detection.

Similar to Y. Chen et al., R. Wang et al. developed one-pot visual RT-LAMP-CRISPR (opvCRISPR), which combines a one-pot assay based on mineral oil separation between the nucleic acid amplification and CRISPR/Cas12a detection steps. The authors used blue-light naked-eye detection, as employed by the two groups mentioned above. Wang et al. used RT-LAMP, which occurs at 65°C at the bottom of the tube, while the CRISPR/Cas12a complex is maintained on the lid and separated by mineral oil (Wang R. et al., 2021). Although one-pot assays are superior to two-pot assays due to the lower potential for contamination, one must consider the possibility of the reagent on the lid evaporating or mixing with the reagent underneath in order to avoid any interference with the assay specificity.

Another group proposed the use of dual crRNAs to increase detection. These researchers added divalent cations to improve the sensitivity of this method. They demonstrated that using Mn^2+^ increased the efficiency and, most importantly, the specificity. Thus, they termed this technique manganese-enhanced Cas12a (MeCas12a) (Ma et al., 2020). It has been found that Mn^2+^ helps to stabilize the most stable bond between CRISPR and target DNA (Sundaresan et al., 2017). The addition of Mn^2+^ aided in detecting virus in clinical samples from patients with both SARS-CoV-2 and Middle East respiratory syndrome-related coronavirus (MERS-CoV). These viruses share highly conserved regions in their nucleic acid and differ by only a few single-nucleotide polymorphisms. Therefore, the addition of Mn^2+^ improved the detection of both viruses, without false detection of one virus over the other. Notably, the authors showed that Mn^2+^ helped improve the efficiency of *Lachnospiraceae bacterium* (LbCas12a), but not *Acidaminococcus* sp. (AsCas12a) in this paper. For AsCas12a, Mn^2+^ activated Cas12a even in the absence of target DNA, increasing the nonspecificity. This detection technology was not designed for POCT applications, but this higher-specificity method can be applied to future studies for POCT.

Guo et al. developed CASdetec (CRISPR-assisted detection), which has a LoD of 10 RNA copies per *µ*L (Guo et al., 2020). This study was unique because of their careful study of each platform parameter. As a prime example, the authors found an optimal combination of crRNA concentration and ssDNA-FQ reporter length with the best fluorescence signal; in contrast, other studies are lacking in this regard. Similar to others, Guo et al. attempted to develop a one-pot reaction by placing the CRISPR complex in the cap of the tube, which carries the risk of premixing prior to the reaction, as stated above. As another unique aspect, the authors based their detection method on the RdRp locus of the virus, in contrast to the other studies. Other studies showed that RdRp tends to mutate more than other regions of the virus nucleic acid (Eskier et al., 2020), which could interfere with the detection sensitivity. Moreover, this study lacked clinical data, which would be useful to obtain.

Another group modified a component of the CRISPR-based assay to improve its sensitivity. This method was called ENHANCE (enhanced analysis of nucleic acids with crRNA extensions) and was developed to find the optimal length of crRNA extension to increase the collateral cleavage activity of Cas12a (Nguyen et al., 2020). The authors discovered that a 3’ DNA with 7-mer extensions showed the brightest fluorescence for some orthologs of Cas12a. They studied the crystal structure of Cas12a to form a hypothesis regarding crRNA extension and its influence on collateral cleavage activity. Interestingly, this study produced results that contradicted those of Ma et al., 2020. The authors found out Mn^2+^ inhibited the LbCas12a activity, in contrast to Mg^2+^, which increased the activity. This contradiction should be further studied with the same controls to determine the reason behind the two different results. This discrepancy may have arisen because the crRNAs were designed for different targets. All of the assays mentioned in this section are summarized below in **Table 1**.

**Table 1:** Nucleic acid detection of SARS-CoV-2 using CRISPR/Cas12-based assays.

Assay Name	CRISPR Protein	Nucleic Acid Amplification	Target Region	LoD^x^	Clinical Samples	Type of clinical sample	Other major components	Testing time	One pot *vs.* two pots	References
DETECTR	Cas12a	RT-LAMP	N and E gene	10 RNA copies/*µ*L	11	Nasopharyngeal and oropharyngeal	ssDNA probe, lateral flow assay, 62°C water bath.	30-40 minutes	two pots	Broughton et al., 2020
CRISPR-FDS	Cas12a	RT-RPA	ORF1ab and N gene	2 RNA copies	29	Nasal swab	Fluorescent probe, fluorescence plate reader, 42°C water	50 minutes	two pots	Huang et al., 2020
iSCAN	Cas12a for 2 pots or Cas12b for one pot assay	RT-LAMP	N and E gene	10 RNA copies/reaction	31	Nasopharyngeal	ssDNA-FQ reporter or lateral flow assay, 62°C water bath and fluorescence plate reader	1 hour	both	Ali et al., 2020
Y. Chen et al. assay	Cas12a	RT-LAMP	ORF gene, N gene and E gene	20 RNA copies/reaction	10	Unknown	ssDNA probes, mineral oil, portable 3D printing or smartphone to detect fluorescence, 65°C water bath	40 minutes	one pot	Chen Y. et al., 2020
CRISPR/Cas12a-NER	Cas12a	RT-RAA	E gene	10 RNA copies	31	Unknown	ssDNA-FQ reporter, 39°C water bath, blue light with a wavelength of 485 nm	45 minutes	two pots	Wang X .et al., 2020
AIOD-CRISPR	Cas12a with 2 crRNAs	RPA	N gene	5 RNA copies	28	Nasal swab	ssDNA-FQ reporter, UV and blue LED light	40 minutes	one pot	Ding et al., 2020
opvCRISPR	Cas12a	RT-LAMP	S gene	5 RNA copies	50	Nasopharyngeal	ssDNA reporter, mineral oil, air column, blue LED light, 65°C water bath	45 minutes	one pot	Wang R. et al., 2021
MeCas12a	Cas12a with 2 crRNAs	RT-RAA	E gene	5 RNA copies	24	Nasopharyngeal	ssDNA-FQ reporter, Mn^2+^, 39°C water bath, wavelength of 485 nm light.	45 minutes	two pots	Ma et al., 2020
CASdetec	Cas12b	RT-RAA	RdRp locus	10 RNA copies/*µ*L	0	NA	ssDNA-FQ reporter, blue LED light	1 hour	one pot	Guo et al., 2020
ENHANCE	Cas12a with 3’DNA7-modified crRNA	RT-LAMP	N gene	3-300 RNA copies	0	NA	FITC reporter, lateral flow assay, 63°C water bath, Mg^2+^	30 minutes	two pots	Nguyen et al., 2020

^x^unit is different between studies. Some did not mention the units.

37°C degree water bath can be replaced by body heat, and therefore was not included.

NA, not applicable.

## Cas13 Effector-Based COVID-19 Diagnostic Tool

Even before Cas12 was used as a detection method, scientists had been exploring the potential of Cas13 as a diagnostic tool. One of the first Cas13 nucleic acid detection methods was developed by Zhang and colleagues in 2017. This method, called Specific High-Sensitivity Enzymatic Reporter UnLOCKing (SHERLOCK), has been adapted by many different labs for various uses, such as the detection of the white spot syndrome virus in shrimp (Sullivan et al., 2019), the Ebola and Lassa viruses (Barnes et al., 2020), and malaria (Lee et al., 2020). As previously mentioned, Cas13 is a unique protein that can target an RNA sample, rather than a DNA sample like Cas12. This is advantageous because the SARS-CoV-2 is a single-stranded RNA virus (Carter et al., 2020). In fact, the investigators who originally developed SHERLOCK received FDA approval for COVID testing under emergency use purposes (Guglielmi, 2020). This was a big step toward the development and improvement of CRISPR-based detection and opened the door for many other efforts. SHERLOCK uses RPA technology with Cas13 and can detect aM sensitivity in synthetic virus samples (Gootenberg et al., 2017). However, minimal clinical data have been presented. Moreover, SHERLOCK requires a plate reader to detect the fluorescence reading, which is not ideal for POCT. Thus, the same researchers developed SHERLOCK version 2 (SHERLOCK v2) to detect multiple diseases from a sample and make the technology more applicable to POCT (Gootenberg et al., 2018).

Another lab has demonstrated that SHERLOCK can detect SARS-CoV-2 clinical samples (Patchsung et al., 2020). This group used RT-RPA to amplify the viral gene and designed their primers around the S, N, and ORF1ab regions. The results showed that detection of the S gene using SHERLOCK was the most sensitive, both with fluorescence readouts and a lateral flow strip, with a LoD of 42 RNA copies per reaction. We note that this sensitivity is not as good as the Cas12 nucleic acid detection mentioned earlier. Interestingly, this group had access to a large number of clinical samples and used 154 nasopharyngeal samples to test the S region of the virus. Their detection method was able to detect 100% (73/73) of the negative samples using either the fluorescence readout or lateral-flow strip readout; detection of the positive samples was 96% (78/81) and 88% (71/81), respectively. This reduced sensitivity may be due to a low amount of viral RNA in the samples. The researchers then implemented SHERLOCK as a quick (70 minute) pre-operative screening tool for patients in Thailand entering the operating room. However, the samples were all negative, due to overall low numbers of COVID cases in this country following nationwide lockdown to reduce viral spread. As a result, this method has not yet been tested in patients who are positive for SARS-CoV-2.

Another group developed a CRISPR-COVID test by targeting the ORF1ab and N genes of SARS-CoV-2 and demonstrated better sensitivity and specificity with ORF1ab detection (Hou et al., 2020). The LoD of this method was 7.5 RNA copies per reaction, which is an improvement. This group also presented clinical data with good specificity and sensitivity.

Recently, researchers developed another Cas13a-based detection method using a similar design approach as others, except without the nucleic acid amplification step (eliminating a step in **Figure 1C**). This technology made it possible to detect the virus in 30 minutes, although the LoD was 100 RNA copies/μL (Fozouni et al., 2021). This technology utilizes Cas13a and a quenched fluorescent RNA reporter, making it very simple and unique. The tool combines three crRNAs to efficiently detect the N and E gene of SARS-CoV-2, even though the samples are not amplified. To make their assay closer to POCT, these researchers used a mobile phone camera as a portable plate reader to eliminate the need for a fluorescence plate reader. They tested this intriguing idea on five clinical samples and demonstrated a LoD of 200 RNA copies/μL when using a mobile phone. Despite the potential of this approach, the current study has many flaws. The sample size of five clinical samples is small, and 200 even 100 RNA copies/μL is too high for a clinical test. Arnaout et al. showed in their preprint article that increasing LoD by a factor of 10 could increase the rate of false negatives by 13% (Arnaout et al., 2020). Perhaps combining this triple crRNA approach with isothermal amplification and a lateral-flow assay for measurement would improve the sensitivity and make substantial strides toward POCT.

Finally, researchers have designed Cas13-based, rugged, equitable, scalable testing (CREST) to be cost effective and widely accessible to the public. In this article, they opted for the miniPCR and P51 fluorescence visualizer, which are both portable and small devices. This method is cheaper and more effective at detecting the virus compared with other PCR methods (Rauch et al. 2021). Notably, a miniPCR is compatible with mobile devices, creating the possibility that clinics could acquire a patient’s data using a mobile phone and upload the data directly to the cloud. However, this assay is a ‘two-pot’ assay and must be performed by highly trained scientists. All the Cas13 effector-based COVID-19 diagnostic tools mentioned in this section are summarized in **Table 2**.

**Table 2:** Nucleic acid detection of SARS-CoV-2 using CRISPR/Cas13 and Cas9-based assays.

Assay Name	CRISPR Protein	Nucleic Acid Amplification	Target Region	LoD^x^	Clinical Samples	Type of clinical sample	Other major components	Testing time	One pot *vs.* two pots	References
Patchsung et al. assay	Cas13a	RT-RPA	S gene	42 RNA copies/reaction	154	Nasopharyngeal and throat swab	ssRNA reporter, Lateral flow assay, RNase-responsive RNA reporter, 42°C water bath	1 hour	two pots	Patchsung et al., 2020
CRISPR-COVID	Cas13a	RT-RPA	ORF1ab	7.5 copies/reaction	114	Unknown	ssRNA reporter, 42°C water bath, fluorescence plate reader	40 minutes	two pots	Hou et al., 2020
Fozouni et al. assay	Cas13a with 3 crRNAs	No amplification	N and E gene	100 copies/*µ*L	5	Nasal swab	ssRNA-FQ reporter, mobile phone to detect fluorescence or plate reader	30 minutes	one pot	Fozouni et al., 2021
CREST	Cas13a	MiniPCR bio	N1 and N2 region	10 RNA copies/*µ*L	159	Nasopharyngeal and oropharyngeal	ssRNA-FQ reporter, fluorescence LED visualizer	50 minutes	two pots	Rauch et al. 2021
FELUDA	Cas9	RT-PCR or RT-RPA	N gene	Unknown	Unknown	Unknown	FAM reporter, Lateral flow assay, 95°C water bath	45 minutes	two pots	Azhar et al., 2020

^x^unit is different between studies. Some did not mention the units.

37°C degree water bath can be replaced by body heat, and therefore was not included.

## Cas9 Effector-Based COVID-19 Diagnostic Tool

The Cas9 effector is well known for its applications in genome editing, though not for its ability to detect nucleic acids. This is because Cas9 lacks collateral activity. However, one group used Francisella novicida Cas9 (FnCas9) to develop an FnCas9 editor-linked uniform detection assay (FELUDA) to detect the presence of SARS-CoV-2 in their preprint article (Azhar et al., 2020). Because FnCas9 has a high specificity for mismatches, it can improve the assay’s specificity over the use of Streptococcus pyogenes (SpCas9), which is a very popular Cas9 ortholog used for gene editing (Chen F. et al., 2017). The findings using FELUDA are intriguing; however, this approach has not yet been compared to the other nucleic acid detection assays mentioned above (**Tables 1** and **2**). The results did not mention a LoD or how many clinical samples were used for testing **(** **Table 2** **)**. It will be intriguing to see more data in the future, especially if this assay ultimately shows significantly higher specificity than the Cas12- and Cas13-based nucleic acid detection methods. More importantly, for a CRISPR-based diagnostic tool to reach the market, it must have as much if not more specificity and sensitivity than the current detection method.

## Microfluidic Device for COVID-19 Diagnosis by CRISPR

In this section, we review microfluidic diagnostic devices that are beyond lateral flow strips. These approaches are more ‘advanced’ but also more complex. Some quantifiably measure SARS-CoV-2 using digital chip while others use engineering technologies such as microfluidic devices to detect the virus.

As the majority of the CRISPR-based diagnostic tools mentioned above lack the ability to measure the quantity of the virus, one group developed a RApid DIgital CRISPR Approach (RADICA) in their preprint article to quantifiably measure SARS-CoV-2 like RT-qPCR. This one-pot assay works in one hour using RPA, CRISPR-Cas12a, an ssDNA-FQ reporter, chip-based digital PCR, and a Clarity™ Reader for fluorescence detection (Wu et al., 2020). This device can analyze the proportion of positive to negative signals by partitioning a patient’s sample into 10,000 portions on a chip. The specificity is good, and the device can even differentiate SARS-CoV-2 from a similar coronavirus. It is important that the device can perform reverse transcription, because SARS-CoV-2 is an RNA virus. However, since the RPA/RT-RPA can be initiated at room temperature, their reaction mixture needs to be prepared on ice and loaded into chips within one minute to minimize premature target amplification.

To prevent undesired premature amplification and accurately quantify nucleic acid, another group developed a digital warm-start CRISRP (WS-CRISPR) assay, which is only initiated at above 50°C. In this preprint article, they use reverse transcription dual-priming mediated isothermal amplification (RT-DAMP), Mg^2+^, and pyrophosphatase (PPase) (Ding et al., 2021). The researchers take advantage of the mediation role of pyrophosphatase and phosphorothioate primers to develop a low-temperature RT-DAMP assay. Also, they optimize each concentration and the amount of each component to achieve higher specificity. This assay is clinically validated by quantitatively detect 35 clinical samples (32 swab samples and three saliva samples), showing 100% agreement with RT-PCR results. However, the detection process is labor intensive as it relies on fluorescence microscopy, and ImageJ software to count the number of positive spots on the chip compared to using a fluorescence reader like RADICA. By applying a fluorescence-automated reader, this device could be improved and simpler in the future.

**Figure 2:**
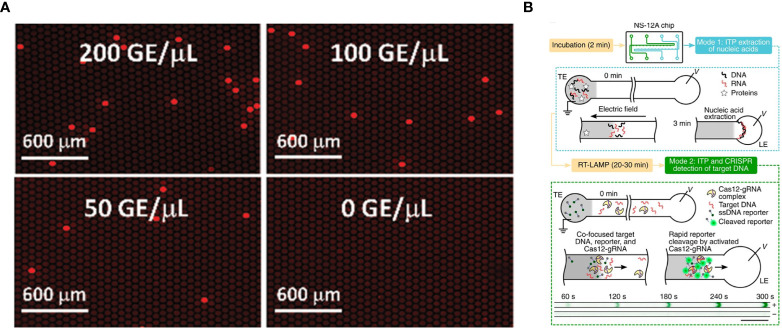
SARS-CoV-2 detection on microfluidic diagnostic devices using CRISPR/Cas technology. **(A)** Fluorescence images of digitization-enhanced CRISPR/Cas-assisted one-pot virus detection (deCOVID) on microfluidic digital chips for SARS-CoV-2 detection (Digital CRISPR/Cas-assisted assay for rapid and sensitive detection of SARS-CoV-2 by Park et al., 2020 is licensed under CC BY 4.0). **(B)** Working principle of isotachophoresis-CRISPR (ITP-CRISPR) assay. The microfluidic chip has two channels for ITP extraction of nucleic acids (mode 1) and ITP–CRISPR detection (mode 2). In the mode 1, when an electric field is applied, nucleic acids selectively focus within the electromigrating LE–TE interface, leaving behind impurities. After off-chip RT-LAMP of ITP-extracted nucleic acids, in mode 2, ITP is used to detect target DNA by using a CRISPR-Cas12. A positive sample shows a strong fluorescent signal, while negative control not (Electric field-driven microfluidics for rapid CRISPR-based diagnostics and its application to detection of SARS-CoV-2 by Ramachandran et al., 2020 is licensed under CC BY 4.0).

With a similar goal of achieving faster, more accurate quantitative analysis, digitization-enhanced CRISPR/Cas-assisted one-pot virus detection (deCOVID) was developed using a similar approach as the devices mentioned above (**Figure 2A**). This device is faster but requires more components like WS-CRISPR (Park et al., 2020). The researchers used 0.1% Tween-20 to reduce the viscosity of the reaction mix to be compatible with digital PCR. They also used bovine serum albumin (BSA) to improve the reaction performance by reducing the adsorption of enzymes to the reaction tube. One interesting finding was that deCOVID’s reaction time stayed constant even when there was a small amount of RNA present in the sample, unlike other assays. Future work could simplify the device, and possibly incorporate the use of a mobile phone instead of fluorescence microscopy.

All the devices mentioned above lack the ability to extract and purify nucleic acid from nasopharengeal or any other clinical samples. Rather, these devices rely on samples that are already extracted and ready to use. This is a big remaining hurdle because nucleic acid extraction and purification usually takes an hour and is an important step for accurate detection (Ramachandran et al., 2020). To this end, one group developed isotachophoresis-CRISPR (ITP-CRISPR) using microfluidics and an on-chip electric field (**Figure 2B**). ITP can be applied for nucleic acid extraction and purification from biological samples and the on-chip electric field enables the detection of SARS-CoV-2 by CRISPR. ITP-CRISPR is remarkably fast, with the nucleic acid extraction and purification and detection of the sample taking less than 40 minutes (Ramachandran et al., 2020). Another advantage of this assay is a reasonable reaction volume requirement, making it cost effective for the reagent that is used. Using a large number of clinical samples, ITP-CRISPR was able to detect 30 out of 32 positive samples (93.8%) and 32 out of 32 negative samples (100%). But this assay required off-chip manual steps for RT-LAMP and sample lysis. Off-chip steps potentially increase the risk of contamination, especially if tasks are not performed by someone who is well trained. Nonetheless, it is important to consider nucleic acid extraction, as it is the initial step for fully integrated nucleic acid detection. Several microfluidic diagnostic devices mentioned in this section are summarized in **Table 3**.

**Table 3:** SARS-CoV-2 detection on microfluidic diagnostic devices using CRISPR technology.

Assay Name	CRISPR Protein	Nucleic Acid Amplification	Target Region	LoD^x^	Clinical Samples	Type of clinical sample	Other Major components	Testing time	One pot *vs.* two pots	References
RADICA	Cas12a	RPA	N gene	LoQ*-2.2 DNA copies/*µ*L	0	NA	ssDNA-FQ reporter, chip-based digital PCR, Clarity™ Reader for detecting fluorescence, 42°C incubation, ice	1 hour	one pot	Wu et al., 2020
WS-CRISPR	Cas12a	RT-DAMP	N gene	5 RNA copies/*µ*L in the reaction	35	Saliva and unknown clinical swab	ssDNA-FQ reporter, QuantStudio 3D digital PCR, Mg^2+^, pyrophosphatase, 52°C incubation, fluorescence microscopy with camera, ImageJ software	50 minutes	one pot	Ding et al., 2021
deCOVID	Cas12a	RT-RPA	N gene	1 GE^^^/*µ*L	4	Unknown	ssDNA reporter, QuantStudio 3D digital PCR, 0.01 mg/mL BSA, 0.1% Tween-20, 42°C incubation, fluorescence microscopy with camera, QCapture software, ImageJ software	30 minutes	one pot	Park et al., 2020
ITP-CRISPR	Cas12	RT-LAMP	N and E gene	10 copies/*µ*L	64	Nasopharyngeal	ssDNA reporter, 62°C incubation, lysis buffer, on-chip ITP, fluorescence microscope, sourcemeter, camera,	40 minutes	two pots	Ramachandran et al., 2020

^x^unit is different between studies. Some did not mention the units.

37°C degree water bath can be replaced by body heat, and therefore was not included.

*LoQ-Limit of quantification.

^^^GE-genome equivalent.

NA, not applicable.

## Challenges and Perspectives

Faster nucleic acid-based molecular detection methods are needed during this pandemic, but it is especially important that these detection methods are POCT so that they can be easily applied in developing countries and to people who live hours away from clinics and hospitals. Faster and more accurate detection methods could improve the speed of contact tracing and help control the pandemic (Koetter et al., 2020). Reduced costs and waiting times for results would also be beneficial to both national economies and patients’ medical bills. Many groups have designed assays that are faster, easier to use, and more applicable to POCT of SARS-CoV-2. RT-qPCR is time consuming, and requires specific temperatures, bulky instruments, and highly trained scientists. However, it is not yet possible to replace RT-qPCR due to the trade-off with other technologies between being POCT friendly and having reduced sensitivity. All the emerging CRISPR approaches detailed in this paper must be tested using clinical data from different sources, such as blood, saliva, and oropharyngeal specimens, in order to prove their specificity, sensitivity, and duration. The assays will also need to be tested on clinical samples of SARS, MERS, and other coronaviruses to prove that they can distinguish between similar viruses despite shared conserved genomic regions. Moreover, not all the assays have shown aM sensitivity, which will be required before replacing RT-qPCR.

It appears that the performance of the CRISPR protein can vary depending on which organism it is isolated from, (Gootenberg et al., 2018; Ali et al., 2020; Nguyen et al., 2020). For example, addition of Mn^2+^ does not improve the sensitivity of all Cas12a proteins, because of the differing function depending on which organism the Cas12a is isolated from (Ma et al., 2020). There have also been contradictions between some studies. One study mentioned that Mn^2+^ improved the sensitivity of LbCas12a, whereas another study stated that it inhibited the activity of LbCas12a (Ma et al., 2020; Nguyen et al., 2020). It is important to choose the right organism, the right protein, and the right kind of modification. Several of the studies above also demonstrated that using multiple crRNAs is more effective than using one. There are other proteins similar to CRISPR that have been developed. For example, *Thermus thermophilus (TtAgo)* is guided by ssDNA to cut the target DNA (Song et al., 2020). It does not get limited by the PAM site and uses DNA rather than RNA for the guide, which makes it more versatile. However, it is still new to the nucleic acid detection field, more studies are needed to compare this with CRISPR in terms of its efficiency.

We also note the importance of potential contamination and the presence of ribonuclease (RNase) in preparing an assay to be market ready. Especially in a clinical setting, the presence of RNase can interfere with the result, since they can degrade crRNAs and RNA. Such degradation could lead to a false signal (Patchsung et al., 2020). Therefore, researchers should consider adding RNase inhibitors and implement methods to detect the presence of RNase, such as the assay developed by Patchsung et al. In general, it is imperative to avoid any contamination, especially for assays that will need to be manufactured at large quantities and distributed globally. For these reasons, ‘one-pot’ assays are preferable over ‘two-pot’ assays to avoid the need for opening a tube multiple times.

To improve these assays and bring us closer to POCT, lyophilization of reagents could improve transportation and storage and prevent protein and RNA damage, because protein and RNA are temperature sensitive. Qian et al. also showed that using trehalose and pullulan to dehydrate Cas12a protein makes it possible to store the protein for more than a month at room temperature (Qian et al., 2020).

Even with all these improvements, DNA/RNA extraction is the biggest hurdle for bringing nucleic acid detection to POCT, because few nucleic acid extraction methods are POCT friendly. Not only does the extraction step require an extensive multi-step process, trained technicians and bulky equipment, but most of the extracted DNA/RNA needs to be purified before the amplification step to remove potential inhibitors that could further interfere with the downstream application (Zou et al., 2017). As a result, patients still need to visit a clinic for COVID-19 testing, because nucleic acid extraction must happen before CRISPR can be used on the samples. Complicating things further, the extraction method varies depending on the origin of the samples, because the component that is required is different for each sample. It would be convenient to use raw samples right away for detection without extracting and purifying it, but there are still remaining challenges for accurate diagnostics. Depending on which samples are used, some of the samples need to be diluted, for example blood and mucus samples (Paul et al., 2020). Others such as, saliva or nasopharyngeal samples need enough of the virus or pathogen genetic material present in it to be detected. Without the nucleic acid extraction method, most of the detection assays mentioned above would not be able to meet clinical diagnostic requirements due to reduced sensitivity and specificity.

Additionally, manufacturing the CRISPR protein could be a hurdle in the development of CRISPR-based testing. Currently Cas13 and Cas14 proteins are not commercially available (“Cas13 — Zhang Lab” n.d.). This means that laboratories are currently expressing and purifying these proteins themselves, which could lead to variations between labs. Manufacturing CRISPR using good manufacturing practice (GMP) and making it widely available would hasten the improvement of CRISPR-based detection assays and the time to market.

In summary, CRISPR-based nucleic acid detection is an innovative approach that has progressed rapidly and has the potential for further improvement. Ultimately, if this technology became available in a hospital setting, patients could potentially learn their COVID-19 testing result within an hour at little cost, which would be a great resource during this pandemic. Testing could save lives, because as much as 81% of the people who test positive can be asymptomatic virus carriers, according to testing that was performed in passengers of an Argentinian expedition cruise ship (Ing et al., 2020). It is crucial to perform testing as early as possible to prevent asymptomatic people from spreading to the virus unknowingly to others. If testing is affordable and has a fast turn-around time with good accuracy, then millions of lives could be saved. Following the necessary improvements laid out in this paper, CRISPR-based detection could hopefully become available in a clinical setting or even as an at-home detection kit in the near future. Importantly, this assay would be useful for detecting many other viruses and pathogens at the point of care.

## Author Contributions

UG designed the structure of, wrote, and critically reviewed the manuscript. UG also designed the figures using Biorender.com. CL designed the structure of and critically reviewed the manuscript, and approved its final version. All authors contributed to the article and approved the submitted version.

## Funding

This research was supported, in part, by NIH R01EB023607, R61AI154642, and R01CA214072.

## Conflict of Interest

The authors declare that the research was conducted in the absence of any commercial or financial relationships that could be construed as a potential conflict of interest.
